# Standardized and batch effect–independent technologies enable global collaboration in microbiome research

**DOI:** 10.1093/ismejo/wrag122

**Published:** 2026-05-13

**Authors:** Muzi Ge, Tomoya Maeda, Jingdi Li, Maryam Chaib De Mares, Emmanuel George Kifaro, Gizachew Haile Gidamo, Katsuyuki Shiroguchi, Andrew H Moeller, Zhibin Zhang, Jianshi Jin

**Affiliations:** State Key Laboratory of Animal Biodiversity Conservation and Integrated Pest Management, Institute of Zoology, Chinese Academy of Sciences, 1 Beichen West Road, Chaoyang District, Beijing 100101, P. R. China; College of Life Science, University of Chinese Academy of Sciences, No. 19A Yuquan Road, Shijingshan District, Beijing 100049, P. R. China; Laboratory of Microbial Physiology, Research Faculty of Agriculture, Hokkaido University, Sapporo, Hokkaido 060-8589, Japan; Department of Zoology, University of British Columbia, 6270 University Blvd, Vancouver, BC V6T 1Z4, Canada; Grupo de Biología Molecular Teórica y Evolutiva, Departamento de Biología, Facultad de Ciencias, Universidad Nacional de Colombia, Bogotá 111321, Colombia; Department of Microbiology, Parasitology and Biotechnology, College of Veterinary Medicine and Biomedical Sciences, Sokoine University of Agriculture, P.O. Box 3019, Morogoro 67125, Tanzania; Biotechnology and Bioprocess Centre of Excellence, Addis Ababa Science and Technology University, P.O. Box 16417, Addis Ababa, Ethiopia; Department of Biotechnology, College of Natural and Applied Sciences, Addis Ababa Science and Technology University, P.O. Box 16417, Addis Ababa, Ethiopia; Laboratory for Prediction of Cell Systems Dynamics, RIKEN Center for Biosystems Dynamics Research (BDR), Kobe 650-0047, Japan; Department of Ecology and Evolutionary Biology, Princeton University, Princeton, NJ 08544, United States; School of Ecology, Hainan University, No. 58 Renmin Avenue, Haikou, Hainan 570228, P. R. China; Hainan International One Health Institute, Hainan University, No. 58 Renmin Avenue, Haikou, Hainan 570228, P. R. China; State Key Laboratory of Animal Biodiversity Conservation and Integrated Pest Management, Institute of Zoology, Chinese Academy of Sciences, 1 Beichen West Road, Chaoyang District, Beijing 100101, P. R. China; College of Life Science, University of Chinese Academy of Sciences, No. 19A Yuquan Road, Shijingshan District, Beijing 100049, P. R. China

**Keywords:** global collaboration, microbial transmission, phylosymbiosis, batch effects, benchmarking, standardized technology

## Introduction

**Figure 1:**
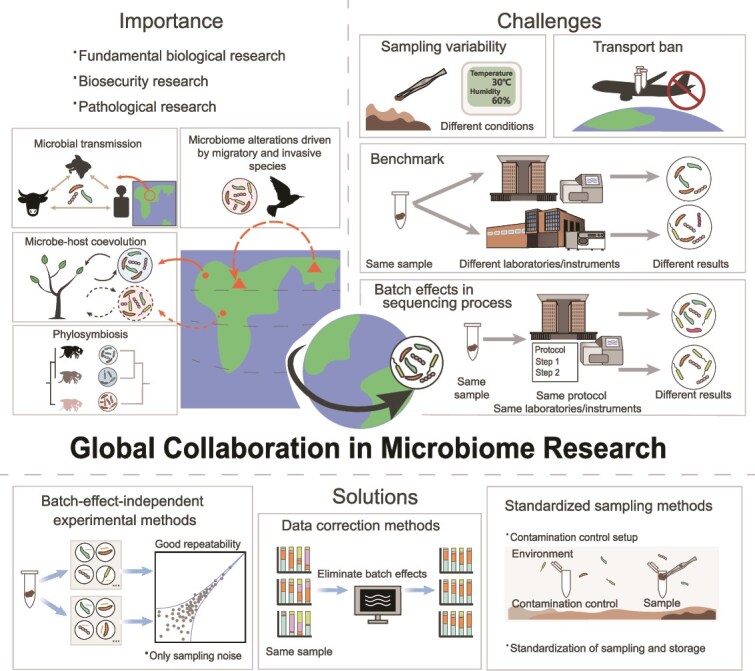
Importance, challenges, and solutions in the global collaboration of microbiome research.

The microbiome is an important subject of research in multiple scientific disciplines because of its ability to shape biogeochemical cycles and regulate both host health and disease states [1]. Whereas traditional microbiome investigations have focused on static characterizations of microbiome–host interactions (e.g. the modulation of host physiology, immune responses, and metabolic pathways [2–4]), recent studies have attempted to investigate the temporal and spatial mechanisms underlying the establishment and dynamic changes in microbiomes (e.g. the variation across hosts and geographic settings and the changes over time within a host or population [5, 6]) (Fig. 1). In addition to the dynamics of the microbiome within its host, the transmission of microbes across different hosts through social interactions, such as direct physical contact and shared environments, has recently attracted increased attention [5]. The transmission of microbial pathogens is a crucial stage in infectious diseases and zoonoses; thus, investigating the mechanisms of microbial transmission is important for improving global public health. Furthermore, large-scale comparative analyses of microbiomes across hosts belonging to different species have provided critical insights into microbe–host interactions from ecological and evolutionary perspectives. For instance, on the basis of metagenomic analyses of the gut microbiota (a narrower term than microbiome, referring to the assemblage of living microorganisms in a defined environment) in wild animals, researchers have identified significant associations between the taxonomic and functional profiles of the microbiota and its host ecological traits (e.g. diet, habitat, and phylogenetic relatedness), providing insights into the ecological and evolutionary mechanisms underlying microbe–host symbiosis [7]. Consortium-driven comparative studies of microbiomes, such as those conducted by Yatsunenko *et al*. [8], the Human Microbiome Project [9], and the Earth Microbiome Project [10], have accelerated microbiome research while also highlighting the need for broader and more internationally representative collaboration. In this perspective, we summarize sources of bias that complicate international collaboration, review recent advances in addressing one potential source of technical bias: batch effects [11–13], and suggest avenues for future work to standardize microbiome profiling across disparate biological systems and research teams.

To study the mechanism of microbial transmission, microbe–host coevolution, or phylosymbiosis, extensive sampling across different geographical regions or host species is needed (Fig. 1) [14–16]. For example, determining the reservoirs and vectors of microbial transmission often requires analyses of samples from multiple host species across different geographical regions [17]. Similarly, interrogating microbe–host coevolution has benefited substantially from analyses of microbiomes from multiple geographically separated and distantly related host species [18]. Furthermore, comparisons of microbial samples obtained from conspecific hosts from different environments can reveal important functional microbes that help the host adapt to extreme conditions, such as extreme temperatures and hypoxia [19, 20]. For some hosts, such as migratory animals and invasive organisms, understanding their associations with their microbiomes necessitates comparisons of host microbiomes in multiple geographic locations, such as before and after migration. Fieldwork across geographically dispersed regions often spans country borders, requiring international collaboration. These international collaborations in microbiome research face significant challenges spanning all stages of study design from sample collection, to processing, to data generation.

Several sources of bias emerge at the stages of sample collection and storage (Fig. 1). First, standardized sampling and storage procedures have yet to be established because the highly variable field sampling environments make it challenging to implement complex and standardized procedures, such as consistent sterilization operations to minimize or eliminate contamination. Second, owing to variations in environmental factors including temperature, humidity, and time of day, even samples collected by using the same procedures may become inconsistent because of environmental influences [21, 22]. Third, variation in the way samples are stored, including the choice of buffers and freezer conditions, can bias downstream estimates of microbiome composition [23, 24]. The impact of these challenges is also likely to vary across study types and sample categories. For example, environmental microbiome studies may be particularly sensitive to variation in field sampling environments [25], whereas fecal or other host-associated studies may be more strongly influenced by sample collection procedures and storage conditions [26]. Importantly, the biases introduced by these sources of sampling variation, metadata inconsistencies, and their interactions have not yet been adequately studied, which complicates establishment of standardized approaches.

In addition to the challenges associated with sample collection, significant difficulties exist in downstream stages of microbiome analysis (Fig. 1). A large body of prior work has shown that different DNA extraction and library preparation methods can have a significant effect on microbiome composition inferred from sequencing, potentially outweighing biological effects of interest [27]. Batch effects have long been a challenge in microbiome research. For example, 16S rRNA gene amplicon sequencing, even when the same protocol is used, may yield significantly different results due to variations in sample storage time, reagent lots, or sequencing platforms, leading to challenges in data reproducibility and comparison [28]. These inconsistencies complicate comparisons across studies, hindering the integration of global datasets. Large cohort studies have further demonstrated that dozens of host and lifestyle covariates materially shape gut community structure and that absolute-abundance calibration (QMP) in 16S rRNA gene amplicon sequencing is often required to make cross-study comparisons interpretable [29]. Similarly, absolute-abundance quantification has also been applied in shotgun metagenomic sequencing [30].

An additional complication is that many countries have strict rules about the transport of biological materials, including bans on certain types of samples and complex approval processes, which makes it impossible to analyze all samples under exactly the same experimental conditions (i.e. in the same laboratory). Therefore, techniques for microbiome analysis that are independent of operators and specific laboratories and free of batch effects need to be developed.

Encouragingly, though no universal solution yet exists, technological advancements are beginning to address these analytical difficulties on multiple fronts (Fig. 1). For instance, to advance the commonly used 16S rRNA gene amplicon sequencing method in this field, we recently developed the BarBIQ (Barcoding Bacteria for Identification and Quantification) method, which identifies bacteria through accurate sequencing of the 16S rRNA genes from each single bacterial cell. Applying this method, we confirmed that the error during repeated measurement of the same sample by BarBIQ came entirely from sampling errors, which maximally reduced the batch effect of the 16S rRNA gene amplicon sequencing–based methods [11, 12]. To date, we have confirmed that BarBIQ has been successfully used in laboratories from different countries, suggesting that this method is not limited to a particular laboratory [11]. Admittedly, this method does not resolve the inherent limitations of the 16S rRNA gene amplicon sequencing itself, such as its ability to detect only a single domain (Bacteria), low detection rates for certain bacterial groups, and poor strain-level resolution. In addition to experimental technical developments, other teams have applied statistical modeling to estimate batch-specific offsets and accordingly adjust microbial abundance data, with the goal of removing systematic variation introduced by batch effects and facilitating cross-study comparability and integrative analyses [13]. While these advances mark the beginning of established global benchmarking methods for microbiome analysis, numerous issues remain in their implementation, as we have summarized in Table 1. Concurrently, standardized benchmarking samples must be developed to assess methodological consistency. Although commercially produced, DNA-based mock microbial communities [31] are used worldwide, standardized whole-cell benchmarking samples have yet to be developed. Importantly, although computational bias-correction methods may be needed for integrating multi-batch data, the development of batch-independent experimental methods represents an important future direction for large-scale microbiome research, given that technical variation is difficult to fully model.

**Table 1:** The problem of methodological bias in microbiome studies.

Source of bias	Current efforts to minimize bias	Existing problems on the bias	Reported effect size
Variation in environmental conditions during sampling	Samples can be preserved promptly after collection (e.g. within a few hours) to minimize alterations to the microbiome; environmental covariates can be incorporated into downstream analyses to account for variations in field conditions [32]	The lack of standardized sampling conditions (e.g. temperature, humidity) for different sample types not only hinders cross-study comparability but also remains an understudied area in methodology development	The variation in microbial relative abundance at the family level caused by pre-freezing delay can be as high as 80% [32]
Storage conditions including different preservation solutions, media, temperature, and time	For DNA analysis, 95% ethanol can be used to reduce batch effects caused by storage in certain sample types (e.g. fecal, saliva, and skin samples) [22]; external spike-in or calibration controls can be used to normalize measurements and correct for technical variations introduced by heterogeneous storage media and buffers [33]; sample storage time can be included as a covariate in the analysis [34]	The efficacy of spike-in controls for storage-related bias correction has not been extensively studied. Despite some investigation into storage duration, optimal time limits for preserving sample integrity have yet to be firmly established	The variation in microbial relative abundance at the phylum level caused by different storage time can be as high as 50% [35]
Microbe lysis and DNA extraction using different kits and protocols	To mitigate inter-laboratory biases, it is recommended to use standardized DNA extraction kits and procedures across all participating laboratories [36]	Standardized protocols or kits suitable for all taxa (e.g. Gram-positive bacteria, spores, fungi, viruses) are insufficiently studied	The variation in the microbiome caused by different DNA extraction kits can be as high as 21.4%; the relative abundances of up to 32% of the detected microbial species can be affected [37]
Batch effects in sequencing-based methods including library preparation and sequencing	The BarBIQ method was developed to mitigate biases from library preparation and sequencing runs, enabling unbiased absolute quantification of microbial composition without batch effects [11]. The ConQuR method was developed to remove batch effects in microbiome data by using conditional quantile regression [13]	While BarBIQ, a 16S sequencing–based method, is limited to profiling bacterial communities, there is currently no counterpart method in metagenomics for all microbial taxa that is free from batch effects	The variation in microbial community structure caused by library preparation is 58.8% [38]
Varying bioinformatics workflows, parameters, and reference databases during data processing	Pipeline-induced biases can be mitigated through three key strategies: harmonizing computational workflows, applying consistent quality-control and taxonomic assignment procedures, and including batch information as a covariate in the analysis [39]	Because analytical outcomes are data dependent, results from a fixed pipeline are subject to variation when applied to different paired samples [28]. While numerous analysis tools and software are available, no consensus exists on a standardized workflow for processing metagenomic sequencing data. Current analytical methods require high-performance computing resources, which limits their applicability in many laboratories	The variation in microbial relative abundance caused by different bioinformatic approaches and database is 59.4% [38]
Missing, inconsistent, or incomplete information in publications	This issue can be mitigated by collecting detailed, standardized metadata [40]. Experimental conditions can be recorded as metadata by standardized sample tracking and processing using barcoded matrix-tube-based workflows [41]. Standardized biological ontologies (e.g. Environment Ontology [ENVO] [42]) and AI-assisted translation tools can be leveraged to harmonize terminology across different languages and overcome language barriers in metadata recording across multinational collaborations	The research community has not yet established standardized criteria for what constitutes sufficient metadata for publication. Terms used to describe the same content may vary across different studies [43]. This necessitates a massive time investment, with researchers often spending 50%–80% of their time on data retrieval and preparation [40, 44]	20.6% of datasets are inaccessible; up to 80% of research time can be lost to data retrieval and cleaning [40, 44]

In conclusion, the complexity and broad nature of microbiome research require broad international collaboration to fully explore microbial diversity and its driving roles across different fields. However, significant barriers, such as differences in experimental conditions, protocols, and regulations, currently hinder such cooperation. To overcome these challenges, we urge that a two-pronged approach be implemented by, first, developing robust, field-ready sampling and preservation standards building on existing protocols [45], and second, adopting analytical methods that are independent of batch effects. Adopting such standards and methods is important for improving the comparability of global data, enabling more accurate modeling, and facilitating international collaboration, which is necessary for comparative microbiome studies. International collaboration will help researchers gain a better understanding of microbiomes and enable a more comprehensive analysis across multiple fields, ultimately contributing to global health and environmental sustainability.

## Data Availability

Data sharing not applicable to this article as no datasets were generated or analyzed during the current study.
